# Potassium Regulation in Medaka (*Oryzias latipes*) Larvae Acclimated to Fresh Water: Passive Uptake and Active Secretion by the Skin Cells

**DOI:** 10.1038/s41598-017-16381-7

**Published:** 2017-11-24

**Authors:** Jiun-Lin Horng, Li-Lu Yu, Sian-Tai Liu, Po-Yen Chen, Li-Yih Lin

**Affiliations:** 1 Department of Anatomy and Cell Biology, Taipei Medical University, Taipei, Taiwan; 2 Department of Life Science, National Taiwan Normal University, Taipei, Taiwan

## Abstract

Molecular mechanisms of Na^+^, Cl^−^, and Ca^2+^ regulation in ionocytes of fish have been well investigated. However, the regulatory mechanism of K^+^ in fishes has been largely unknown. In this study, we investigated the mechanism of K^+^ regulation in medaka larvae acclimated to fresh water. Using a scanning ion-selective electrode technique (SIET) to measure the K^+^ fluxes at skin cells, significant K^+^ effluxes were found at ionocytes; in contrast, significant K^+^ influxes were found at the boundaries between keratinocytes. High K^+^ water (HK) acclimation induced the K^+^ effluxes at ionocytes and suppressed the K^+^ influxes at keratinocytes. The K^+^ effluxes of ionocytes were suppressed by VU591, bumetanide and ouabain. The K^+^ influxes of keratinocytes were suppressed by TAP. *In situ* hybridization analysis showed that mRNA of *ROMKa* was expressed by ionocytes in the skin and gills of medaka larvae. Quantitative PCR showed that mRNA levels of *ROMKa* and *NKCC1a* in gills of adult medaka were upregulated after HK acclimation. This study suggests that medaka obtain K^+^ through a paracellular pathway between keratinocytes and extrude K^+^ through ionocytes; apical ROMKa and basolateral NKCC1a are involved in the K^+^ secretion by ionocytes.

## Introduction

Potassium is a major monovalent cation in the bodies of vertebrate animals. Because of the action of Na^+^/K^+^-ATPase (NKA) on cell membranes, intracellular K^+^ levels are maintained at >20-fold higher than extracellular K^+^ levels. This chemical gradient is critical for establishing the plasma membrane potential. Terrestrial mammals regulate their plasma K^+^ levels within physiological ranges by modulating renal K^+^ excretion into the urine. In mammalian kidneys, K^+^ is freely filtered in the glomeruli, about 90% of the filtered K^+^ in the lumen is reabsorbed in the proximal tube and thick ascending limb, and excess K^+^ is finally secreted in the connecting tubule and cortical collecting duct^1,2^.

Fishes encounter harsh ionic and osmotic gradients derived from their aquatic environments, and the mechanisms for maintaining internal homeostasis are more challenging for fishes compared to terrestrial vertebrates^3^. Freshwater (FW) fishes face ionic losses through their skin and gills, and the loss is balanced by active ion uptake via the gills^3,4^. Ionocytes (also called mitochondrion-rich cells or chloride cells) in the gill epithelium are responsible for Na^+^, Cl^−^, and Ca^2+^ uptake from FW^3^. The molecular mechanisms of Na^+^, Cl^−^, and Ca^2+^ uptake by specific types of ionocytes were revealed in several fish species including zebrafish, tilapia, trout, and medaka^4–7^. Before the full development of the gills, the skin of fish embryos and larvae also develops ionocytes for ion regulation^8^.

Compared to Na^+^, Cl^−^, and Ca^2+^ regulation, K^+^ regulation in fish has been largely unknown in the past few decades. Unlike Na^+^ and Cl^−^ which are maintained at high levels (>100 mM) in plasma, plasma K^+^ levels of fish are usually 4–5 mM^9^. The smaller K^+^ gradient between plasma and the FW environment may limit the passive effluxes of K^+^ through the skin and gills, or even allow K^+^ influxes driven by electrical potentials (transepithelial potentials). Theoretically, high intracellular K^+^ levels do not favor the uptake of K^+^ via a transcellular pathway. It could be a reason why ionocytes have not been speculated to be involved in absorbing K^+^ from FW. In contrast, ionocytes are suggested to secrete K^+^ into FW in recent studies^9,10^. However, it is still unknown how fish take up K^+^ from FW.

In zebrafish and tilapia, a renal outer medullar potassium channel (ROMKa, also called kcnj1 or kir1.1) was identified in a subgroup of ionocytes^9,10^. With a specific antibody, the ROMKa protein was localized to apical membranes of ionocytes in seawater (SW)- and FW-acclimated tilapia (*Oreochromis mossambicus*)^9,11^. High-K^+^ water induced the mRNA expression of ROMKa by gills, suggesting that ROMKa is involved in K^+^ secretion by ionocytes of both FW- and SW-acclimated tilapia^9,11^. ROMK is a K^+^ channel family that exhibits a nonlinear current-voltage relationship, i.e., the inward current is larger than the outward current^12^. In mammals, ROMK was found in apical membranes of tubular epithelial cells from the thick ascending limb, connecting tubule, and cortical collecting duct of the kidneys. In those regions, ROMK is the main route for K^+^ secretion into the lumen^13^.

The Na^+^-K^+^-2Cl^−^ cotransporter (NKCC) is another transport protein that is involved in K^+^ regulation. Two isoforms of NKCC (NKCC1 and NKCC2) were identified in mammals^14,15^. NKCC1 was first found on basolateral membranes of epithelial cells for salt secretion, and thus it is often referred to as the “secretory” isoform^16^. NKCC2 (the “absorptive” isoform) was identified in the thick ascending limb of Henle’s loop and reabsorbs Na^+^, K^+^, and Cl^−^^17^. In SW fishes, basolateral NKCC1 is known to play a critical role in Cl^−^ secretion^3,18^. Interestingly, NKCC1 was also found in the basolateral membrane of ionocytes in FW-acclimated fishes including tilapia^19^, rainbow trout^20^, milkfish^21^, and medaka^7^, although expression levels were not as high as those in SW-acclimated fishes. However, the physiological function of NKCC1 in FW-type ionocytes has not been investigated and is poorly understood. In this study, NKCC1 in basolateral membranes of ionocytes was hypothesized to be involved in K^+^ secretion by medaka in FW.

Application of the scanning ion-selective electrode technique (SIET) to study ion transport has provided accumulating convincing evidence of the mechanisms of ion transport in fish embryos and larvae^22–24^. It was applied to measure H^+^, Na^+^, Cl^−^, NH_4_^+^, and Ca^2+^ fluxes at the surface of ionocytes, keratinocytes, and hair cells in the skin of zebrafish, tilapia, and medaka^23–29^. In this study, K^+^ flux was further analyzed with this electrophysiological technique. Although previous studies suggested that ROMKa mediates K^+^ secretion by ionocytes of tilapia^9,11^ and zebrafish^10^, convincing evidence of K^+^ flux at ionocytes is still lacking. The purpose of this study was to demonstrate K^+^ secretion by ionocytes with the SIET and test the hypothesis that ROMKa and basolateral NKCC1 are involved in the K^+^-secretion mechanism. More importantly, this study attempted to reveal how fish absorb K^+^ from FW. Japanese medaka (*Oryzias latipes*) larvae were used as an animal model because previous studies showed it to be an ideal model for functional studies of ionocytes^22,26,28^.

## Results

### Whole-body ion contents of larvae acclimated to K^+^-free water (KF), normal water (NW) and high-K^+^ water (HK)

**Figure 1:**
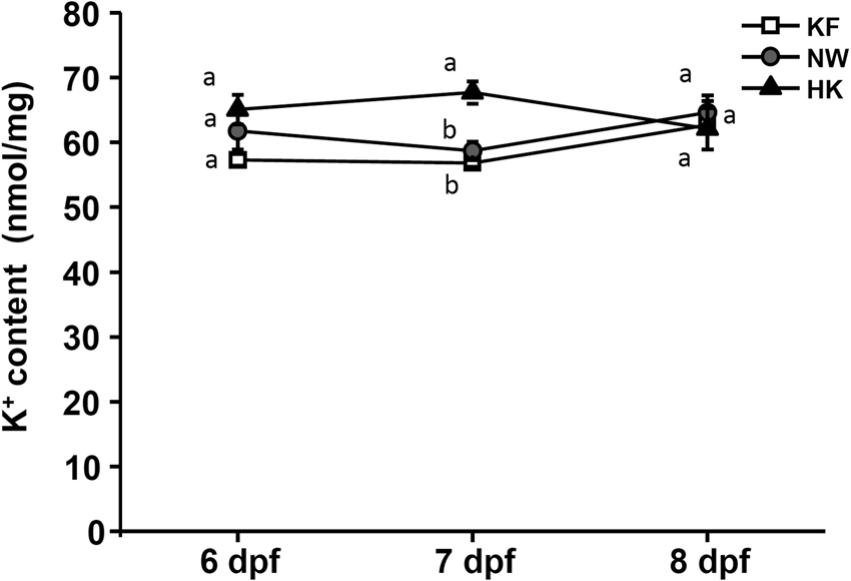
Whole-body K^+^ contents in different stages (6, 7, and 8 days post-fertilization) of medaka larvae acclimated to K^+^-free water (KF), normal water (NW), or high-K^+^ water (HK). Data are presented as the means ± SE (*n* = 5). ^a,b^Different letters indicate a significant difference among the three groups at the same stage (one-way ANOVA, Tukey’s comparison, *p* < 0.05).

To evaluate the capability of K^+^ regulation, medaka embryos were acclimated to three hypotonic media with different K^+^ levels (KF, NW, or HK) from 4–8 days post-fertilization (dpf). All of the larvae survived the acclimation, and no morphological or behavioral abnormalities were observed during the acclimation. Whole-body K^+^ contents of larvae analyzed at 6, 7, and 8 dpf revealed that stable K^+^ contents were maintained during development (Fig. 1). K^+^ contents of the three groups did not significantly differ, except that the content of the HK group was significantly higher than those of the KF and NW groups at 7 dpf (the hatching stage).

### K^+^ fluxes of ionocytes and keratinocytes in the yolk-sac skin

**Figure 2:**
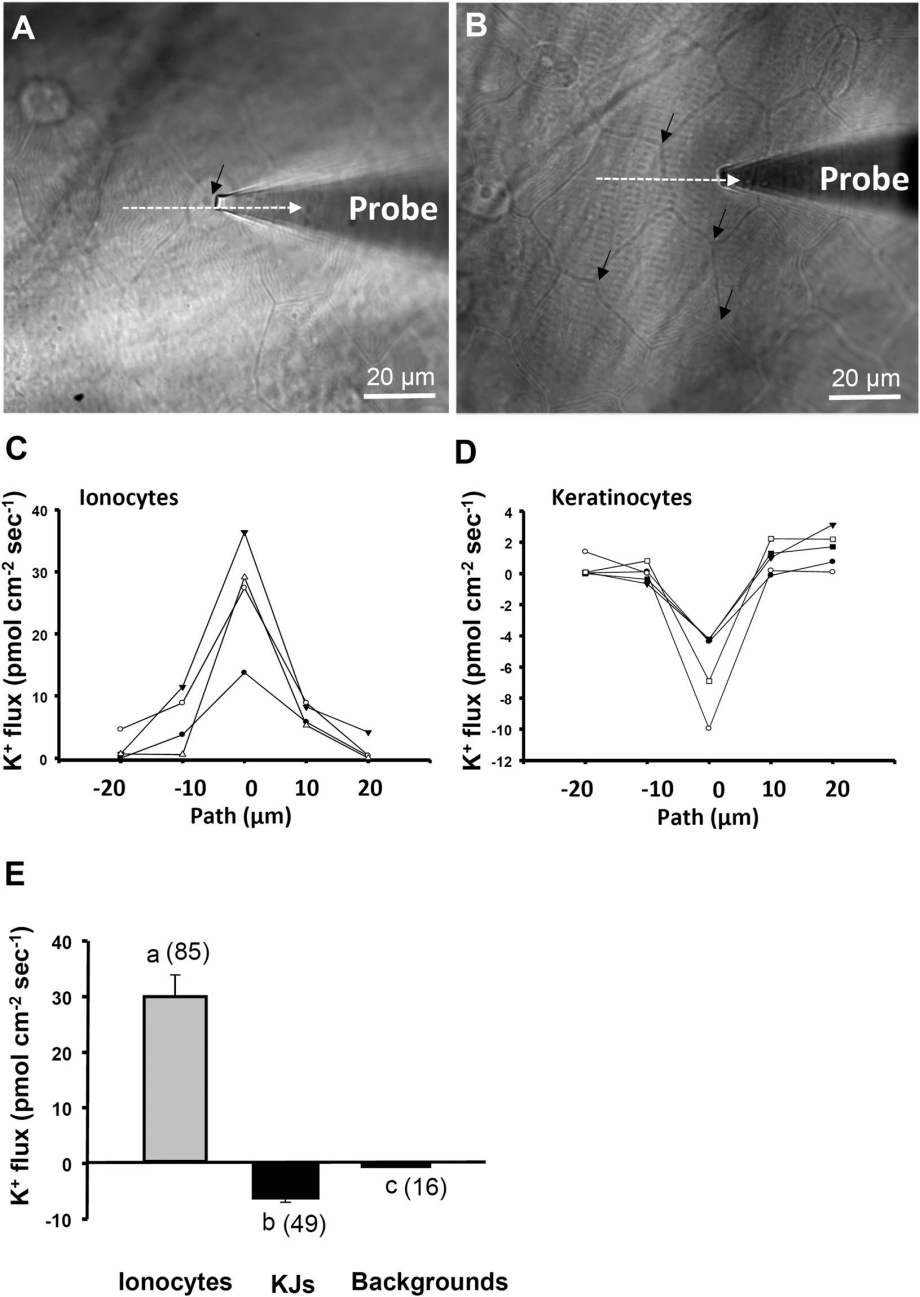
The K^+^ fluxes at ionocytes (ICs) and keratinocytes junctions (KJs) in medaka larvae acclimated to normal water (NW). A “line scan recording” was made by probing at series of locations composing a line (arrow) across the surface of the ionocytes and adjacent keratinocytes (**A**). A “line scan recording” was also performed across the keratinocyte junction (**B**). Four samples of “line scan recording” across the ionocyte were shown in (**C**) and 5 samples of “line scan recording” across the keratinocyte junction were shown in (**D**). To record individual ionocytes, the probe was moved to the apical openings of ionocytes (arrow in A); to record keratinocyte junctions, the probe was moved to the tricellular junction of keratinocyte (arrows in B). The K^+^ fluxes at ionocytes, keratinocyte junctions (KJs), and backgrounds (without fish) were compared. Data are presented as the mean ± SE. Positive values indicate K^+^ effluxes; negative values indicate K^+^ influxes. The number of cells analyzed is shown in parentheses. ^a,b,c^Different letters indicate a significant difference (one-way ANOVA, Tukey’s comparison, *p* < 0.05).

Using the SIET, K^+^ fluxes at specific cells were measured on the surface of skin in medaka larvae acclimated to NW (Fig. 2). Figure 2A shows a “line-scan recording” route across the apical opening of ionocytes and adjacent keratinocytes. Figure 2B shows a “line-scan recording” route across two adjacent keratinocytes. The dashed lines indicate the 40 μm probing routes. When recording across ionocytes, peaks of K^+^ effluxes (positive values) appeared at the apical membrane of ionocytes (Fig. 2C). In contrast, significant inward K^+^ fluxes (negative values) were detected at the boundaries between keratinocytes (Fig. 2D). After recording 85 ionoyctes from 16 larvae, no significant K^+^ influx was found at ionocytes; instead, K^+^ influxes were ubiquitously found at the boundary (tight junction) between keratinocytes suggesting a paracellular pathway of K^+^ influx (Fig. 2E). The “corners” of keratinocyte without ionocytes (tricellular junctions, arrows in Fig. 2B) were chosen as the sites to represent the K^+^ fluxes at “keratinocytes junctions”. Forty-nine corners of keratinocytes were randomly recorded from 16 individuals. The background K^+^ signals recorded in the medium without fish were quite small and negligible (Fig. 2E).

### K^+^ fluxes at ionocytes and keratinocyte junctions in larvae acclimated to KF, NW, or HK

**Figure 3:**
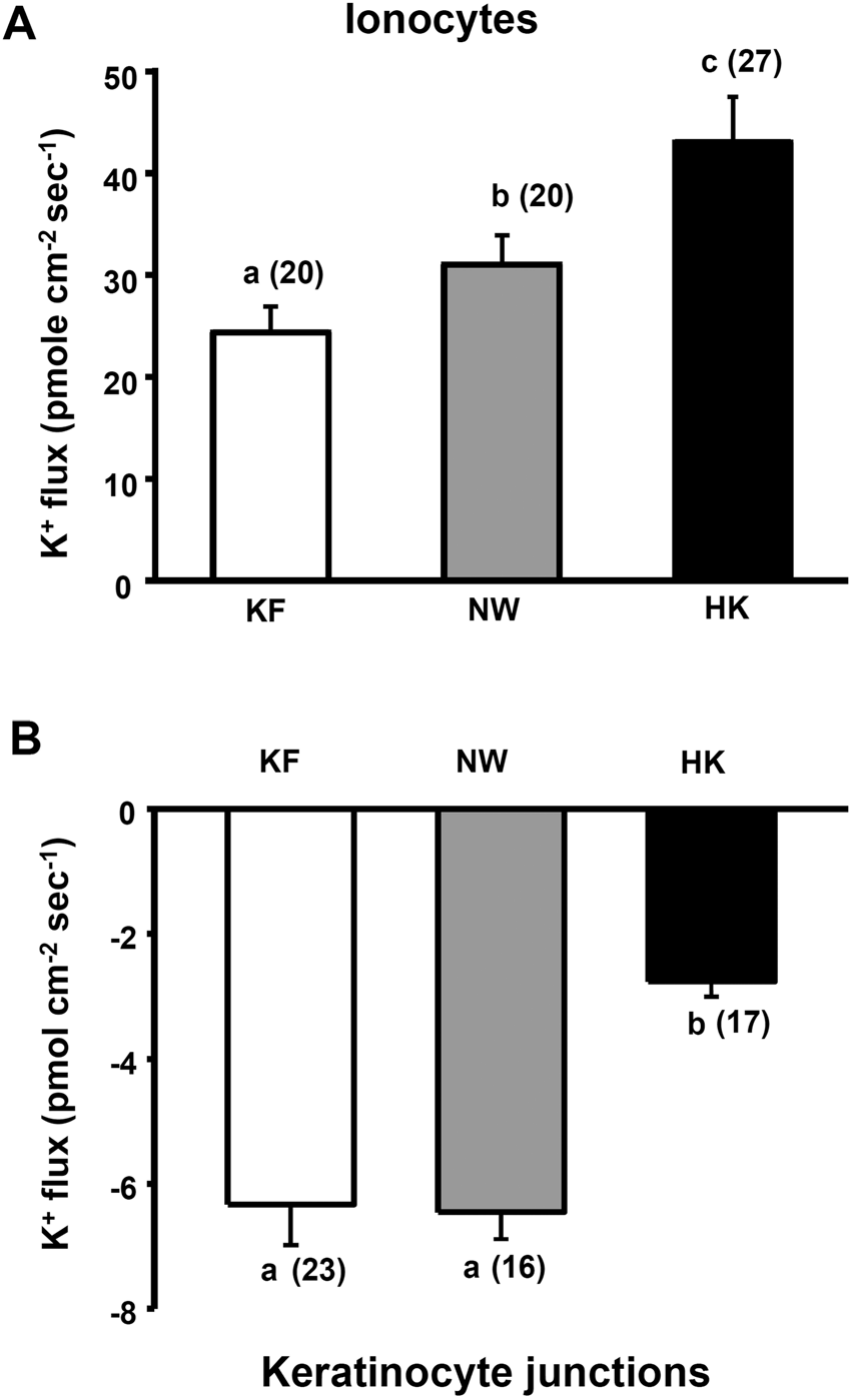
The K^+^ fluxes at ionocytes (**A**) and keratinocyte junctions (**B**) in medaka larvae acclimated to K^+^-free water (KF), normal water (NW), or high-K^+^ water (HK). Data are presented as the mean ± SE. Positive values indicate K^+^ effluxes; negative values indicate K^+^ influxes. The number of cells analyzed is shown in parentheses. ^a,b^Different letters indicate a significant difference (one-way ANOVA, Tukey’s comparison, *p* < 0.05).

The K^+^ fluxes were further measured on medaka larvae acclimated to KF, NW (control group), or HK. The HK acclimation significantly increased the K^+^ effluxes at ionocytes by 35% (Fig. 3A); in contrast, the HK acclimation significantly decreased the K^+^ influxes at keratinocyte junctions by 57% (Fig. 3B). The KF acclimation significantly decreased the K^+^ effluxes at ionocytes by 23% (Fig. 3A) but it did not significantly change the K^+^ influxes at keratinocyte junctions (Fig. 3B).

### Effects of inhibitors on K^+^ effluxes at ionocytes

**Figure 4:**
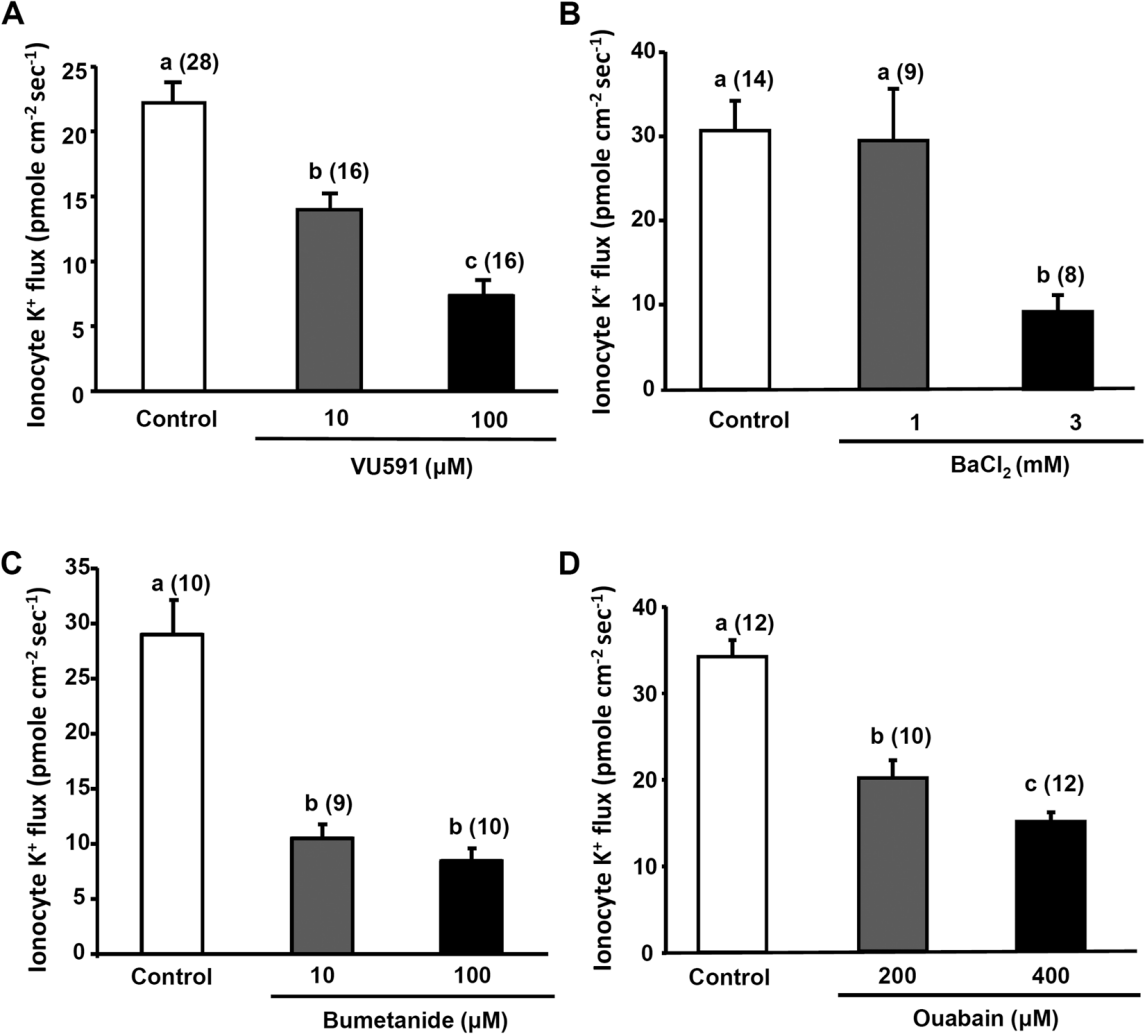
Effects of inhibitors on the K^+^ fluxes at individual ionocytes in larvae acclimated to normal water (NW). VU591 (**A**) and BaCl_2_ (**B**) were used to block the renal outer medullary potassium channel (ROMK), bumetanide (**C**) was used to block the Na^+^-K^+^-2Cl^−^ cotransporter (NKCC), and ouabain (**D**) was used to block the Na^+^ pump. The number of cells analyzed is shown in parentheses. Data are presented as the mean ± SE. ^a,b,c^Different letters indicate a significant difference (one-way ANOVA, Tukey’s comparison, *p* < 0.05).

To examine if ROMK was involved in K^+^ secretion by larvae, a ROMK-specific inhibitor (VU591) was applied to block ROMK. By probing individual ionocytes, K^+^ effluxes had decreased by 40% and 73% after respective treatment with 10 and 100 μM VU591 for 30 min (Fig. 4A). In addition to VU591, a non-specific K^+^ channel inhibitor, Ba^2+^ was also used to block the K^+^ effluxes at ionocytes. After treatment of 3 mM BaCl_2_ for 30 min, approximately 70% of K^+^ efflux was suppressed (Fig. 4B).

Inhibitory effects were also observed in larvae treated with bumetanide (a NKCC inhibitor) or ouabain (a Na^+^ pump inhibitor). The K^+^ effluxes decreased by 64% and 70% after respective treatment with 10 and 100 μM bumetanide for 30 min (Fig. 4C). The K^+^ effluxes decreased by 42% and 54% after respective treatment with 200 and 400 μM ouabain for 30 min (Fig. 4D).

### Effects of inhibitors on K^+^ influxes at keratinocyte junctions

**Figure 5:**
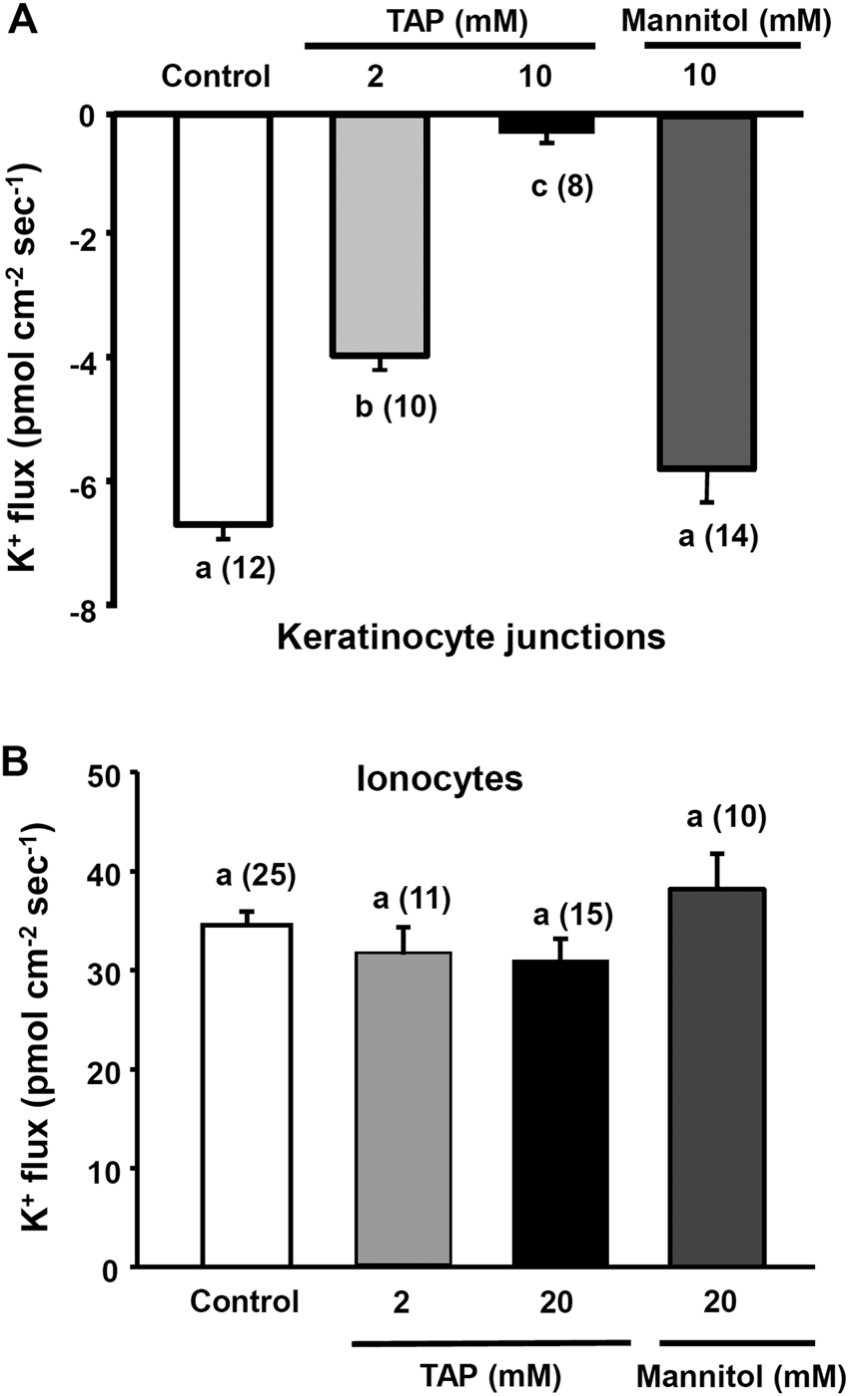
Effects of a tight junction blocker (TAP) on the K^+^ flux at keratinocyte junctions (**A**) and individual ionocytes (**B**) in larvae acclimated to normal water (NW). Mannitol was also used as a negative control. The number of cells analyzed is shown in parentheses. Data are presented as the mean ± SE. ^a,b,c^Different letters indicate a significant difference (one-way ANOVA, Tukey’s comparison, *p* < 0.05).

To further demonstrate that the K^+^ influxes recorded at the keratinocyte junctions were associated with paracellular transports, a tight junction blocker (TAP) was applied to the larvae. After treatment of TAP for 30 min, 2 mM and 10 mM TAP respectively decreased the K^+^ influxes by 42% and 97%. The inhibitory effects were not due to osmotic changes because treatment with 10 mM mannitol did not influent the K^+^ influxes (Fig. 5A). In contrast, TAP (2 and 20 mM) did not affect the K^+^ efflux at ionocytes, suggesting that the K^+^ efflux was not due to passive diffusion through tight junctions (Fig. 5B).

### Effects of high NH_4_^+^ on K^+^ secretion by ionocytes

**Figure 6:**
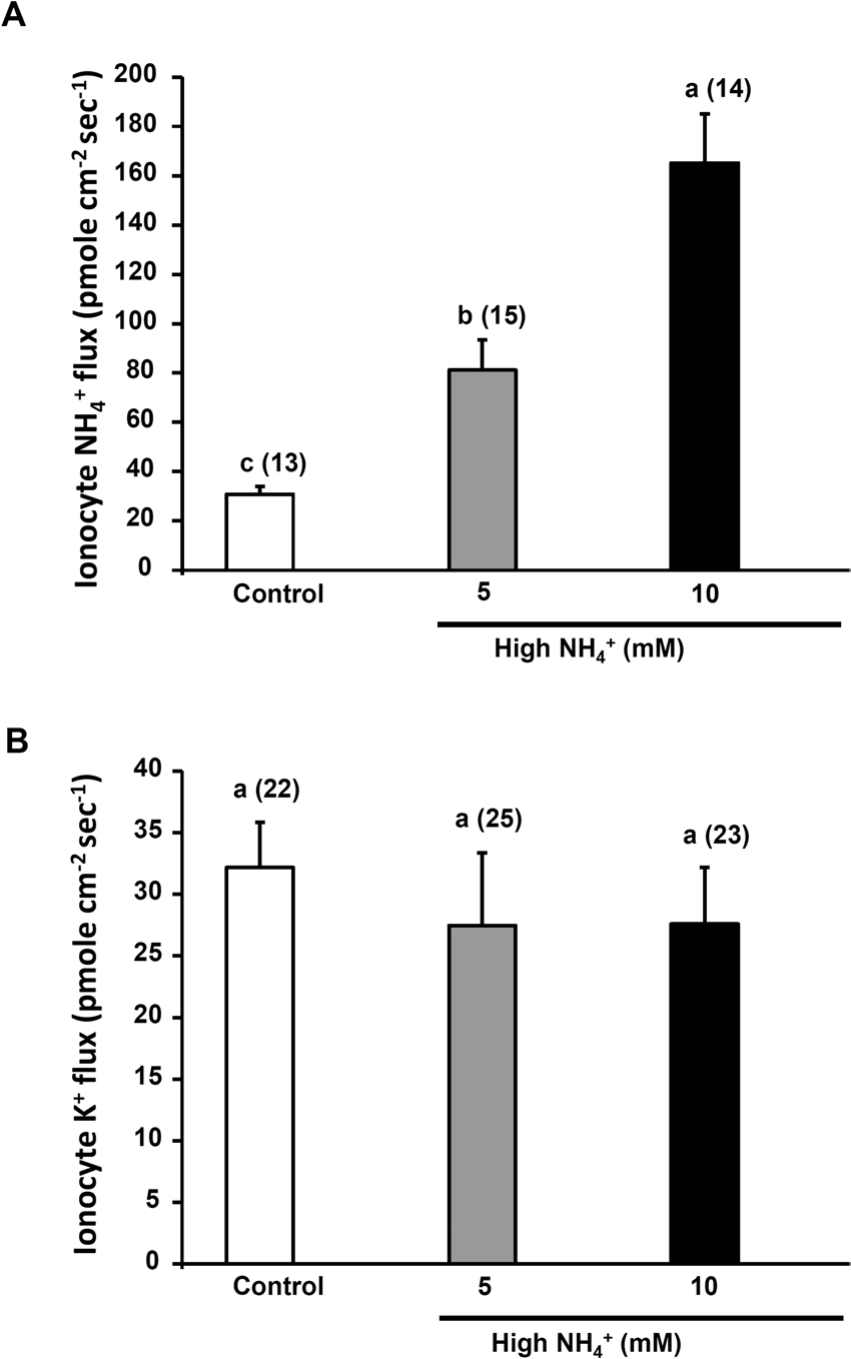
Effects of high ammonium exposure (5 or 10 mM NH_4_Cl) on NH_4_^+^ (**A**) and K^+^ (**B**) fluxes at ionocytes in larvae. Data are presented as the mean ± SE. ^a,b,c^Different letters indicate a significant difference (one-way ANOVA, Tukey’s comparison, *p* < 0.05). The number of cells analyzed is shown in parentheses.

Ionocytes in medaka larvae were revealed to secrete NH_4_^+^ in our previous report^26^. To test if K^+^ secretion was influenced by NH_4_^+^ secretion by ionocytes, both K^+^ and NH_4_^+^ fluxes by ionocytes were measured in larvae exposed to 5 and 10 mM NH_4_Cl for 30 min. We found that NH_4_^+^ fluxes were significantly elevated after NH_4_Cl treatments (Fig. 6A), whereas K^+^ fluxes were not affected by these treatments (Fig. 6B).

### Real-time qPCR analysis of gene expressions by gills of medaka

**Figure 7:**
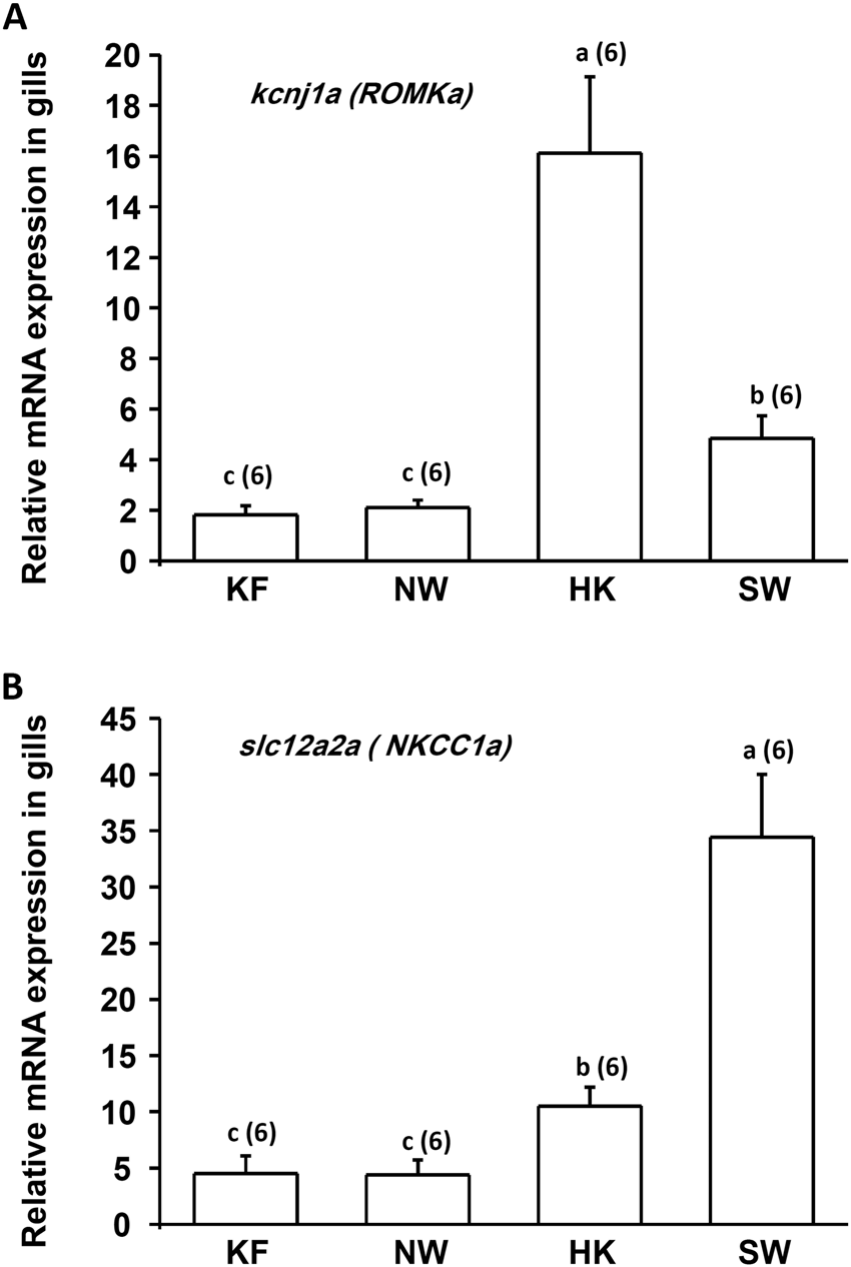
mRNA expressions of *kcnj1a (ROMKa)* and *slc12a2a (NKCC1a)* in gills of adult medaka acclimated to K^+^-free water (KF), normal water (NW), high-K^+^ water (HK), or seawater (SW) for 1 wk. Data are presented as the mean ± SE. ^a,b,c^Different letters indicate a significant difference (one-way ANOVA, Tukey’s comparison, *p* < 0.05).

Real-time qPCR was used to analyze relative mRNA levels of *ROMKa* and *NKCC1a* in gills of adult medaka acclimated to KF, NW, HK, or SW (30 ppt) for 7 d. The SW group was included because *ROMKa* and *NKCC1a* were suggested to be involved in K^+^ secretion by ionocytes of SW-acclimated fish^9^. We found that *ROMKa* was significantly higher in the HK group than in the KF, NW, and SW groups (Fig. 7A). Similarly, *NKCC1a* was significantly higher in the HK group than in the KF and NW groups, whereas *NKCC1a* of the SW group was significantly higher than that of the KF group (Fig. 7B). Both *ROMKa* and *NKCC1a* were induced by HK and SW which contained more K^+^, supporting ROMK and NKCC being involved in K^+^ secretion by ionocytes.

### Localization of the ROMKa in ionocytes of medaka larvae

**Figure 8:**
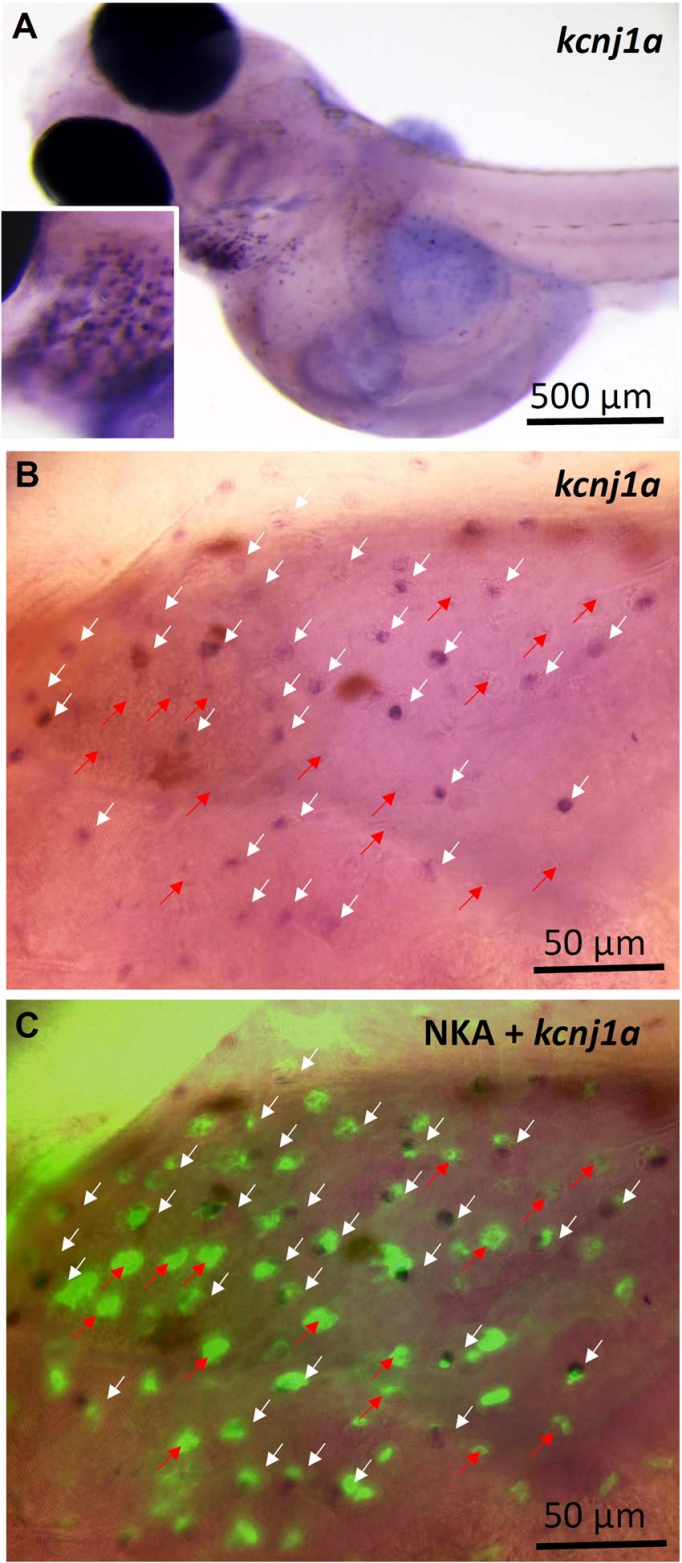
Localization of *kcnj1a* (*ROMKa*) mRNA in ionocytes of medaka larvae acclimated to normal water (NW). (**A**) *kcnj1a* signals were revealed with *in situ* hybridization. A magnified area of the gills is shown in the inset. (**B**) *kcnj1a* signals in yolk-sac skin. (**C**) Double-labeling of *kcnj1a in situ* hybridization and Na^+^/K^+^-ATPase (NKA) immunohistochemistry (green signals). White arrows indicate NKA-labeled ionocytes with *kcnj1a* signals; red arrows indicate NKA-labeled ionocytes without *kcnj1a*.

*In situ* hybridization was used to localize *ROMKa* mRNA in medaka larvae acclimated to NW. Ionocyte-like signals were observed in the skin and gills of larvae (Fig. 8A,B), whereas these signals were absent from the negative control in which a sense probe was used (data not shown). Larvae were further double-labeled with NKA IHC (Fig. 8C) to confirm localization of *ROMKa* signals in ionocytes^7^. The merged image reveals that *ROMKa* was localized to a portion of ionocytes (white arrows in Fig. 8C), but not to other ionocytes (red arrows in Fig. 8C).

## Discussion

Teleost fishes can maintain consistent plasma K^+^ levels. For example, tilapia were shown to maintain their plasma K^+^ concentration within a narrow range (3.8–4.4 mM) after acclimation to environments with various K^+^ concentrations (0.07–17 mM)^9^. To evaluate the capability of K^+^ regulation in embryonic and larval stages, we analyzed whole-body K^+^ contents of medaka larvae acclimated to different K^+^ levels (0 mM in KF, 0.48 mM in NW and 4.8 mM in HK). We found that the content was maintained in a small range of 57–67 nmol/mg. Although a significant difference of the K^+^ content was found between the HK and KF groups at 7 dpf, the difference had recovered by 8 dpf, the post-hatching stage (Fig. 1), indicating that the newly hatched larvae were able to maintain K^+^ homeostasis.

Using the SIET to measure K^+^ fluxes at the skin cells of intact larvae, significant K^+^ effluxes were found at ionocytes (Fig. 2C,E) and the K^+^ effluxes were suppressed by VU591, bumetanide, Ba^2+^, and ouabain (Fig. 4) but not TAP (Fig. 5B). In addition, HK acclimation increased the K^+^ efflux at ionocytes; KF acclimation decreased the K^+^ efflux (Fig. 3A). Taken together, we suggest that the K^+^ effluxes at ionoyctes were not due to leakage of internal K^+^ but channel/transporter-mediated K^+^ secretion. The ROMK, NKA, and NKCC were suggested to be involved in the K^+^ secretion by ionocytes.

Recently, an *in situ* hybridization analysis was used to localize *kcnj1* in skin ionocytes of zebrafish embryos. With two-electrode voltage clamps, *Xenopus* oocytes expressing zebrafish *kcnj1* showed a weak inwardly rectifying current which was inhibited by a non-specific potassium channel inhibitor (Ba^2+^)^10^. Thereafter, two paralogues of ROMK (*kcnj1a* and *kcnj1b*) were found in tilapia^11^. With immunostaining, ROMKa (*kcnj1a*) protein was localized to apical membranes of ionocytes in gills of tilapia acclimated to SW. Using a K^+^ precipitation analysis, K^+^ signals were found at the apical region of ionocytes, and the signals were absent from fish treated with 5 mM Ba^2+^, suggesting that K^+^ is secreted by ionocytes^9^. In addition, ROMKa protein was also localized to apical membranes of ionocytes in FW-acclimated tilapia. ROMKa mRNA levels of gills were upregulated after high-K^+^ FW acclimation, suggesting that ROMKa in gills is also involved in K^+^ secretion by FW-type ionocytes^11^. In this study, we also found two ROMK paralogues (*kcnj1a* and *kcnj1b*) in gills of medaka. *kcnj1a* was dominant and was localized to ionocytes in the gills and skin of larvae (Fig. 8A).

Ba^2+^ and bee venom toxin tertiapin-Q (TPNQ) were previously used to inhibit ROMK; however, they are not ROMK-specific. In mammals, Ba^2+^ can inhibit all Kir channels^30^ and Maxi-K channels^31,32^. TPNQ was found to inhibit G-protein-gated inward-rectifier K^+^ (GIRK1/4) and ROMK channels^33–35^. VU591 used in this study was shown to be a ROMK-specific inhibitor which only inhibits ROMK (Kir1.1) but not other Kir family channels in mammals^36^. VU591 is a newly developed compound and the original report shows that 10 μM VU591 can suppress 86% rat and 89% human ROMK activities in culture cells^36^. In the present study, 10 μM VU591 was shown to suppress K^+^ fluxes of ionocytes by 40% (Fig. 4B).

**Figure 9:**
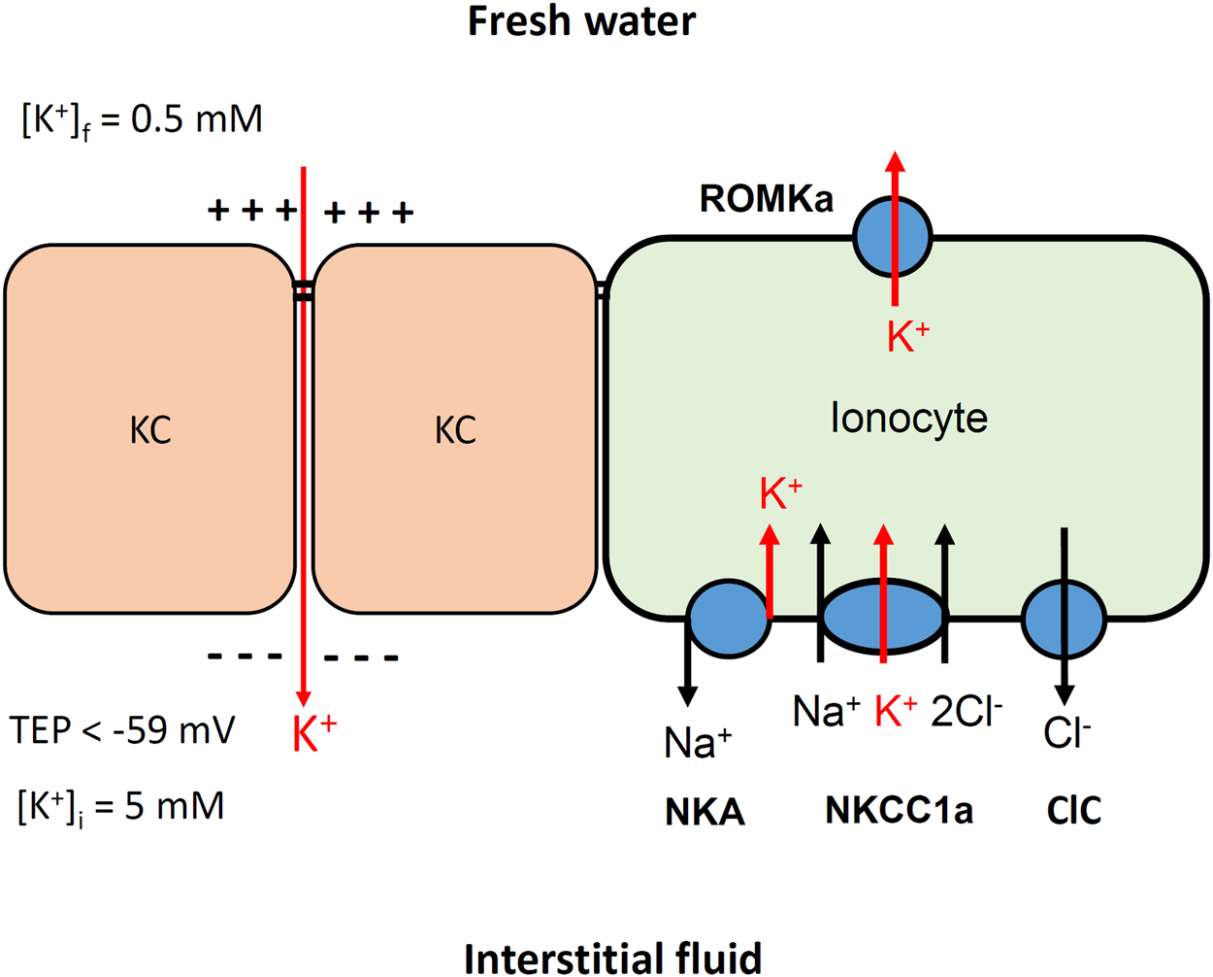
A proposed model for the K^+^ secretion by ionocytes and K^+^ uptake via the paracellular pathways of keratinocytes. See discussion paragraphs for more detail. [K^+^]_f_, freshwater K^+^ concentration; [K^+^]_i_, interstitial K^+^ concentration; TEP, transepithelial potential; ROMK, renal outer medullary potassium channel; NKCC, Na -K -2Cl cotransporter; CLC, Cl^−^ channel; NKA, Na^+^/K^+^-ATPase.

NKCC1 in the basolateral membrane of SW-type ionocytes is well known to play a critical role in Cl^−^ secretion^3,18,37^. However, the function of NKCC1 in FW-type ionocytes was unclear. In a recent study on medaka, NKCC1 was found in the basolateral membrane of FW-type ionocytes (NHE cells) which do not express CFTR^7^. In the present study, K^+^ secretion by ionocytes was suppressed by bumetanide, an NKCC inhibitor. In addition, gene expression of *nkcc1a* in gills was upregulated after HK acclimation (Fig. 7B). These pieces of evidence suggest that NKCC1 is involved in K^+^ secretion by ionocytes. It is known that NKA plays a critical role in driving ion transports by ionocytes. In this study, the K^+^ effluxes at ionocytes were suppressed by ouabain indicating that NKA is involved in K^+^ secretion by ionocyte. Taken together, a model for K^+^ secretion by ionocytes was proposed in this study (Fig. 9). NKA and NKCC1 in the basolateral membrane of ionocytes move K^+^ from interstitial fluid into ionocytes and ROMK in the apical membrane facilitates K^+^ diffusing out of ionocytes. Redundant Cl^−^ in ionocytes can diffuse out of the cell via a basolateral Cl^−^ channel (CLC)^38–40^.

Although ROMK localization in the apical membrane of ionocyte was not successful in this study, it was demonstrated in ionocytes of FW-acclimated tilapia^11^. In addition, ROMK is also expressed in the apical membrane of renal K^+^-secreting cells in mammals^1^. In zebrafish embryos, ROMK (*kcnj1*) mRNA was localized to a subtype of ionocytes which express NKAα1a.4 (NKA.4)^10^, and this subtype was further named K^+^-secreting cells^10,41^. In FW-acclimated tilapia, ROMKa was localized to a subtype of ionocytes which expresses NHE3 and NKA^11^. In this study, ROMKa mRNA was localized to ionocytes in the gills and yolk-sac skin. However, we found that ROMKa mRNA signals were localized to a portion of ionocytes (Fig. 8), implying the existence of a K^+^ secretion-specific subtype of ionocytes. On the other hand, the ROMKa mRNA signals were not robust, the expression pattern may simply represent different expression levels of ROMKa.

The mRNA expression of ROMKa in gills was induced by HK water (Fig. 7A), suggesting that ROMKa is important for K^+^ secretion. In addition, it is an interesting finding that medaka expressed more ROMKa transcript in HK than in SW even though SW contains more K^+^ than does HK (Fig. 7A). Our assumptions are: (1) medaka produced more NKCC1 and NKA in ionocytes to secrete salt in SW, and the abundant NKCC1 and NKA thus provided a stronger driving force to transport K^+^ across the basolateral membrane of ionocytes; (2) K^+^ might be able to pass through the leaky junction between ionocytes and adjacent accessory cells in SW (i.e. Na^+^ secreting pathway).

Other K^+^ channels like Maxi-K, KCC1, and KCC4 were found in gills of tilapia, although their expressions were not altered by high K^+^ acclimation^9^. Their function might also be associated with K^+^ transport in gills. Further investigation of those proteins is required to fully understand K^+^ regulation in fishes.

In early studies, NH_4_^+^ was suggested to compete with K^+^ for transmembrane transport because they have similar ionic radii. For example, the extracellular K^+^-binding sites of NKA and NKCC were suggested to bind NH_4_^+^ for transmembrane NH_4_^+^ transport^42^. In this study, we used both K^+^- and NH_4_^+^-selective probes to measure K^+^ and NH_4_^+^ fluxes at ionocytes and found that high NH_4_^+^ exposure enhanced NH_4_^+^ secretion as reported previously^22,23,25,43^ but did not influence K^+^ secretion by ionocytes (Fig. 5). This result indicates that K^+^ secretion is not associated with NH_4_^+^ secretion by ionocytes in medaka larvae even though both K^+^ and NH_4_^+^ were secreted by ionocytes.

More importantly, this study shows for the first time that K^+^ could be passively absorbed via the tight junction of keratinocytes in medaka larvae. To our knowledge, passive uptake of K^+^ by freshwater fish has not been reported. By using the SIET, K^+^ influxes were found at the junction of keratinocytes and the influxes could be blocked by a tight junction blocker (TAP). TAP was used to block paracellular transport in various epithelia of animals including the colon^44,45^, the ileum^46^, the gall bladder^47^, and the urinary bladder^48^. In fish, TAP was used to block paracellular Na^+^ movement across the opercular membrane^49^.

After HK acclimation, not only K^+^ efflux was enhanced but also K^+^ influx was suppressed suggesting that medaka larvae could regulate both K^+^ efflux and influx in response to high K^+^ water (Fig. 3). However, KF acclimation did not alter the K^+^ influx but only suppressed the K^+^ efflux, suggesting that regulation of K^+^ efflux might be the major way to maintain K^+^ balance in medaka subjected to low K^+^ water (Fig. 3).

Although the K^+^ concentration gradient between freshwater and interstitial fluid may not favor the passive influx of K^+^, a negative transepithelial potential (TEP; water side as 0 mV) may be able to counter the concentration gradient and cause K^+^ influx through tight junctions. TEP is an important parameter in understanding transport mechanisms of the epithelial cells because TEP and the concentration gradient of the relevant ion determining the true electrochemical gradients. The TEP is positive in SW teleosts (water side as 0 mV), whereas it is negative in most FW teleosts^50–52^. The opercular epithelia from FW-acclimated killifish exposed to apical FW exhibited TEP of −40 to −65 mV^53–55^. Studies in intact SW-acclimated killifish showed that TEP was +23 mV, but changed to −39 mV immediately after transfer to FW^56^. Those negative TEPs may drive the paracellular uptake of K^+^ in FW fish. According to the Nernst equation, TEP lower than −59 mV is able to counter passive leakage of K^+^ if the inside of fish has 10 times more K^+^ than the outside (for example, 0.5 mM K^+^ in water and 5 mM K^+^ in plasma; Fig. 9). Further studies with Ussing chambers or voltage clamping techniques are required to demonstrate our assumption in the future.

## Conclusion

This study used a non-invasive technique to demonstrate for the first time that K^+^ can be passively uptake via the paracellular pathway of keratinocytes and actively secreted by ionocytes in the skin of medaka larvae in FW. The apical ROMKa and basolateral NKCC1 and NKA are involved in the mechanism of K^+^ secretion by ionocytes.

## Materials and Methods

### Experimental animals

Mature Japanese medaka were reared in circulating tap water at 27–29 °C with a photoperiod of 14 h of light and 10 h of dark. The females spawned every day, and fertilized egg clusters were collected from the belly of a female and rinsed with tap water to remove the sludge and separate the clusters into single eggs. The eggs were incubated in an incubation chamber at 27 °C with a photoperiod of 14 h of light and 10 h of dark. Medaka embryos usually hatched at 7–8 days post-fertilization (dpf), and newly hatched larvae were used for the experiments. During the experiments, larvae were not fed, and the media were changed daily to maintain water quality. The experimental protocols were approved (no. 95013) by the National Taiwan Normal University Animal Care and Utilization Committee, and were carried out in accordance with the approved guidelines.

### Acclimation experiments

All of the incubating and acclimating solutions were prepared by adding salts (Sigma-Aldrich, St. Louis, MO) to redistilled water. Normal water (NW) contained (in mM) 0.5 NaCl, 0.2 CaSO_4_, 0.2 MgSO_4_, 0.16 KH_2_PO_4_, and 0.16 K_2_HPO_4_ (pH 6.8, adjusted with NaOH or HCl). High-potassium water (HK) contained (in mM) 0.5 NaCl, 0.2 CaSO_4_, 0.2 MgSO_4_, 0.16 KH_2_PO_4_, 0.16 K_2_HPO_4,_ and 4.32 KCl (pH 6.8, adjusted with NaOH or HCl). KCl was used to raise K^+^ content because Cl^−^ did not influence K^+^ secretion. In a preliminary test, we raised the Cl^−^ level of water with NaCl (4.32 mM) and found that K^+^ secretion was not elevated after acclimation. Potassium-free water (KF) contained (in mM) 0.5 NaCl, 0.2 CaSO_4_, 0.2 MgSO_4_, 0.16 NaH_2_PO_4_, and 0.16 Na_2_HPO_4_ (pH 6.8, adjusted with NaOH or HCl). Seawater (SW) was prepared by adding proper amounts of sea salt (Instant Ocean, Aquarium System, Mentor, OH). Thirty-ppt SW contained approximately (in mM) 462 Na^+^, 521 Cl^−^, 9.4 K^+^, 9.4 Ca^2+^, 52 Mg^2+^, and 23 SO_4_^−^ (pH 8.3). For NW, KF, HK, and SW acclimation, fertilized eggs were incubated in NW for the first 2 days and then transferred to NW, KF, or HK for 6 days. For NW and HK acclimation of adult medaka, fish were transferred to 5-liter tanks containing NW or HK for 1 week. During the acclimation experiment, the fish were not fed to avoid the effect of electrolytes in the feed, and the acclimation solutions were changed daily to maintain water quality.

### Measurement of whole-body K^+^ contents in medaka larvae

Medaka larvae were briefly rinsed with deionized water and dried in an oven overnight, and 30 individuals were pooled as one sample and weighed. HNO_3_ (70%) was added to samples for digestion at 60 °C overnight. Digested solutions were diluted with double-deionized water, and the total K^+^ contents were measured with an atomic absorption spectrophotometer (model Z-8,000; Hitachi, Tokyo, Japan). Standard solutions of K^+^ measurements from Merck (Darmstadt, Germany) were used to make the standard curve.

### Scanning ion-selective electrode technique (SIET)

The SIET was used to measure K^+^ activities at the skin and ionocyte surfaces of larvae. Glass capillary tubes (no. TW 150-4, World Precision Instruments, Sarasota, FL) were pulled on a Sutter P-97 Flaming Brown pipette puller (Sutter Instruments, San Rafael, CA) into micropipettes with tip diameters of 3–4 μm. These were then baked at 120 °C overnight and coated with dimethyl chlorosilane (Sigma-Aldrich) for 3 h. The micropipettes were backfilled with a 1-cm column of electrolytes and frontloaded with a 50-μm column of liquid ion-exchange cocktail (Sigma-Aldrich) to create an ion-selective microelectrode (probe). The following ionophore cocktails (and electrolytes) were used: potassium ionophore I - cocktail B (100 mM KCl) and NH_4_^+^ ionophore I cocktail B (100 mM NH_4_Cl). To calibrate the ion-selective probe, the Nernstian property of each microelectrode was measured by placing the microelectrode in a series of standard solutions (0.1, 1, 10, and 100 mM KCl for the K^+^ probe; 0.1, 1, and 10 mM NH_4_Cl for the NH_4_^+^ probe). By plotting the voltage output of the probe against log[K^+^] and log[NH_4_^+^]values, a linear regression yielded a Nernstian slope of 59.1 ± 0.5 (*n* = 10) for K^+^ and 58.6 ± 0.8 (*n* = 10) for NH_4_^+^.

### Measurement of K^+^ fluxes at specific skin cells

The SIET was performed at room temperature (26–28 °C) in a small plastic recording chamber (radius: 2 cm; height: 0.5 cm) filled with 2 ml of the recording medium. The recording medium contained 0.5 mM NaCl, 0.2 mM CaSO_4_, 0.2 mM MgSO_4_, 0.16 mM KH_2_PO_4_, 0.16 mM K_2_HPO_4_, 300 µM MOPS buffer, and 0.3 mg/l ethyl 3-aminobenzoate methane-sulfonate (MS-222, Sigma-Aldrich). The pH of the recording medium was adjusted to 7.0 ± 0.3 by adding a NaOH or HCl solution. Before the measurement, an anesthetized larva was anesthetized with the recording medium for 3 min and then positioned in the center of the chamber with its lateral side contacting the base of the chamber. To record the surface K^+^ flux at ionocytes, the microelectrode was moved to a position about 2 μm above the apical surface of the ionocyte. Voltage differences in micro volts (μV) were recorded by probing orthogonally to the surface at 10-μm intervals. Ten replicates of single-point recordings were made of a cell, and the median value of the repeats was used to calculate the K^+^ flux of the cell.

Previous reports^25,26,57–59^ explained the calculation of ionic fluxes. Voltage differences obtained from ASET software were converted to a concentration gradient using the following equation: ΔC = C_b_ × 10^(Δ*V*/*S*)^ − C_b_, where ΔC (µmole 1^−1^ cm^−3^) is the concentration gradient between two points, C_b_ (µmole 1^−1^) is the background ion concentration, Δ*V* is the voltage gradient obtained from ASET software, and *S* is the Nernst slope of the electrode. The concentration gradient was subsequently converted to ionic flux using Fick’s law of diffusion: *J* = *D*(ΔC)/ΔX, where *J* (pmole cm^−2^ s^−1^) is the net flux of the ion, *D* is the diffusion coefficient of the ion (in NW: 1.96 × 10^−5^ cm^2^ s^−1^ for K^+^), ΔC (pmole cm^−3^) is the concentration gradient, and ΔX (cm) is the distance between the two points.

### Treatments with inhibitors

The ROMK inhibitor (VU591 and BaCl_2_), NKCC inhibitor (bumetanide), NKA inhibitor (ouabain) and tight junction inhibitor (2,4,6-triaminopyrimidine, TAP) were obtained from Sigma-Aldrich. A stock solution of VU591 was prepared by dissolving it into dimethyl sulfoxide (DMSO, Sigma-Aldrich). The final concentration of DMSO in the working solutions (including the control group) was 0.1%. BaCl_2_ stock solution was prepared by dissolving it in redistilled water. Stock solutions of bumetanide were prepared by dissolving it in ethanol (Sigma-Aldrich). The final concentration of ethanol in the working solutions (including the control group) was 0.1%. TAP was directly dissolved with NW to the final concentrations. Before SIET recordings, the larvae were incubated in 1 ml of NW medium with different concentrations of inhibitors for 30 min. 0.1% DMSO or ethanol was added to the control group. After incubation, the larvae were transferred to the recording medium that contained no inhibitor. The inhibitor was not added to the recording medium to prevent alteration of the selectivity of the microelectrodes. In preliminary tests, VU591 (100 μM) was found to increase the background voltage of recording medium by 2.6% and decrease the Nernstian slope to 54.5 ± 0.5 (n = 5). Other inhibitors used in this study did not significantly alter the voltage or slope of calibration curve.

### Preparation of RNA

To obtain a sufficient quantity of RNA, adult medaka gills isolated from three individuals (40–50 mg) were pooled as one sample. Samples were homogenized in 0.5 ml Trizol Reagent (Invitrogen, Carlsbad, CA), and DNA contamination was removed with Dnase I (Promega, Madison, WI). Total RNA was purified by a MasterPure™ RNA Purification Kit (Epicentre Biotechnologies, Madison, WI). The amount of total RNA was determined using a NanoDrop 1000 spectrophotometer (Thermo Scientific, Wilmington, DE). All RNA pellets were stored at −20 °C for less than 1 week.

### Real-time quantitative polymerase chain reaction (qPCR) analysis

For complementary (c)DNA synthesis, 5 μg of total RNA was reverse-transcribed in a final volume of 20 μl containing 0.5 mM dNTPs, 2.5 μM oligo(dT) primer, 250 ng of random primers, 5 mM dithiothreitol (DTT), 40 units of an RNase inhibitor, and 200 units of SuperScript III RT (Invitrogen) for 1 h at 55 °C, followed by incubation at 70 °C for 15 min. mRNA expressions of the target genes were measured by a real-time qPCR with the ABI StepOne Plus sequence analysis system (Applied Biosystems, Foster City, CA) in a final volume of 10 μl, containing 5 ng of cDNA, 50 nM of each primer, and LightCycler® 480 SYBR Green I Master (Roche, Mannheim, Germany). Specific primers for all genes (*kcnj1a*, F: 5′-ACGGAGACCTGACGTGGCAAGA-3′ and R: 5′-AGAGCTACAGCACCTGGGCAGA-3′; *slc12a2a*, F: 5′-TCTGGTGGCTGTTTGATGATG-3′ and R: 5′-AGGCAGGCTTATGACGATGA-3′) were designed using Primer Premier software (vers. 5.0; PREMIER Biosoft International, Palo Alto, CA). All real-time qPCRs were performed as follows: 1 cycle of 50 °C for 2 min and 95 °C for 10 min, followed by 40 cycles of 95 °C for 10 s, 60 °C for 10 s, and 72 °C for 10 s (the standard annealing temperature of all primers). PCR products were subjected to a melting-curve analysis, and representative samples were electrophoresed to verify that only a single product was present. A no-template control reaction was conducted with sterile water to determine the levels of background. The standard curve of each gene was checked in a linear range with ribosomal protein L7 (*rpl7*) as an internal control. The calculation of relative mRNA levels was based on the comparative Ct method^60^.

### RNA probe synthesis

A fragment of ROMKa (*kcnj1a*, Ensemble ID: ENSORLG00000013560) was obtained by a PCR and inserted into the pGEM-T Easy vector. Specific forward and reverse primers were 5′-CCTTCCTGGCTGACTTCTG-3′ and 5′-GCTCCAATGAGGGACTGTATGA-3′. The inserted fragments were amplified with the T7 and SP6 primers by a PCR, and the products were respectively used as templates for *in vitro* transcription with T7 and SP6 RNA polymerase (Roche, Penzberg, Germany). Digoxigenin (DIG)-labeled RNA probes were examined using RNA gels and a dot blot assay to confirm their quality and concentrations.

### *In situ* hybridization and immunohistochemistry (IHC)

Medaka larvae were anesthetized on ice and then fixed with 4% paraformaldehyde in a phosphate-buffered saline (PBS) solution at 4 °C overnight. Afterwards, samples were washed with diethylpyrocarbonate (DEPC)-PBST (PBS with 0.1% Tween-20) several times (for 10 min each). After a brief rinse with PBST, embryos were incubated with hybridization buffer (HyB: 50% formamide, 5× saline sodium citrate (SSC), and 0.1% Tween 20) at 65 °C for 5 min and with HyB containing 500 μg/ml yeast transfer (t)RNA at 65 °C for 4 h before hybridization. After overnight hybridization with 100 ng/ml DIG-labeled antisense or sense RNA probes, embryos were serially washed with 50% formamide-2× SSC (at 65 °C for 20 min), 2× SSC (at 65 °C for 10 min), 0.2× SSC (at 65 °C for 30 min, twice), and PBST at room temperature for 10 min. Afterwards, embryos were reacted with an alkaline phosphatase-coupled anti-DIG antibody (diluted 1: 5000) and then treated with nitro blue tetrazolium (NBT) and 5-bromo-4-chloro-3-indolyl phosphate (BCIP) for the alkaline phosphatase reaction.

For double-staining, *in situ*-hybridized samples were subjected to IHC. After PBST washing, samples were incubated with 3% bovine serum albumin (BSA) and 5% normal goat serum for 2 h to block nonspecific binding. Some samples were then incubated overnight at 4 °C with an α5-monoclonal antibody against the α-subunit of the avian Na^+^-K^+^-ATPase (diluted 1: 500) (Developmental Studies Hybridoma Bank, University of Iowa, Ames, IA). After PBST washing for 20 min, samples were further incubated in goat anti-mouse immunoglobulin G (IgG) conjugated with Alexa Fluor 488 (Molecular Probes, Carlsbad, CA) for 2 h at room temperature. Subsequently, samples were washed with PBST and stored in PBST at 4 °C in a dark box. Finally, images were obtained using a fluorescence microscope (Axioplan 2 Imaging, Carl Zeiss, Oberkochen, Germany).

### Statistical analysis

Data are expressed as the mean ± standard error (SE; with *n*, the number of larvae or specific cells). Values from each condition were analyzed using a one-way analysis of variance (ANOVA) followed by Tukey’s pairwise comparisons. Significance was set at *α* level of 0.05.
